# Direct access from general practice to transvaginal ultrasound for early detection of ovarian cancer: a feasibility study

**DOI:** 10.1080/02813432.2021.1922831

**Published:** 2021-06-07

**Authors:** Marie-Louise Ladegaard Baun, Margit Dueholm, Hanne Nørgaard Heje, William Hamilton, Lone Kjeld Petersen, Peter Vedsted

**Affiliations:** a Department of Public Health, Aarhus University, Aarhus, Denmark; b Research Centre for Cancer Diagnosis in Primary Care, Research Unit for General Practice – Aarhus, Aarhus, Denmark; c Department of Gynaecology and Obstetrics, Aarhus University Hospital, Palle Juul-Jensens, Aarhus, Denmark; d General practice, Aarhus, Denmark; e University of Exeter Medical School, University of Exeter, Exeter, UK; f Department of Gynaecology and Obstetrics, Odense University Hospital, Odense, Denmark

**Keywords:** Denmark, early diagnosis, general practice, ovarian neoplasms, ultrasonography

## Abstract

**Objective:**

To investigate the feasibility of providing general practitioners (GPs) direct and fast referral access to transvaginal ultrasound (TVUS).

**Design:**

A prospective cohort study.

**Setting:**

A total of 232 Danish general practices in parts of the Central Denmark Region.

**Subjects:**

Women aged ≥40 years who consulted their GP for vague and non-specific symptoms (*n* = 479).

**Main outcome measures:**

The feasibility assessment included the GPs’ referral rate, indications for referral, management of test results, and findings from TVUS.

**Results:**

A total of 479 women were referred to TVUS. The examinations revealed abnormalities in 104 (21.7%) women. Additional investigations were needed in 68 (14.2%) women of whom seven (1.5%) underwent major surgery. No case of ovarian cancer was diagnosed during the study period or the 6-month follow-up. However, three (0.6%) women with an abnormal transvaginal ultrasound were diagnosed with urogynecological cancer; this yielded a PPV of 4.4% (95% confidence interval: 1.5–12.2) and an NPV of 100.0% (95% confidence interval: 96.7–100.0) for urogynecological cancer.

**Conclusion:**

Providing GPs with direct access to transvaginal ultrasound was feasible; 80% of the investigated women were referred back to the GP, 14% were further investigated, 0.6% were diagnosed with urogynecological cancer, and 1.5% had major procedures performed without complications.

**Implications:**

Direct access to TVUS could be an important pathway to ensure fast evaluation of women presenting with vague non-specific symptoms of potential ovarian cancer. Future studies should explore the patient experience, cancer outcomes, and health economics issues.

**KEY POINTS**
   **Current awareness**  • GPs have no fast referral option for women presenting with vague non-specific symptoms that could indicate underlying ovarian cancer.   **Key findings**  • We offered GPs direct and fast referral access to TVUS; 51.7% of practices used the opportunity.  • The GPs referred 479 women to TVUS; 104 had an abnormal TVUS and 68 needed additional investigations.  • Seven women underwent major surgery, leading to three cases of urogynecological cancer. No woman had a false negative TVUS result.

## Introduction

Ovarian cancer (OC) is the most deadly of all gynecological cancers, and the incidence in Denmark is among the highest in the world [1]. The stage at diagnosis is an important prognostic factor; the 5-year survival is poor in women diagnosed with advanced stages (15–30%) compared to women diagnosed with early stages (70–88%) of OC in Denmark [2]. Two in three women are diagnosed with advanced stages [2], and prolonged time to diagnosis has been suggested to be a contributing factor.

A standardised cancer patient pathway (CPP) for OC was implemented in Denmark in 2009 to reduce the time from the first symptom presentation in general practice until treatment [3]. A list of OC-associated symptoms is provided in the CPP, including abdominal distention/bloating, reduced appetite/malaise, urinary frequency, constipation/ileus, fatigue and dyspnea. If a symptom is presented to the general practitioner (GP) and an abdominopelvic mass is identified, urgent referral through the CPP is encouraged. However, only approximately 31–36% of OC cases are diagnosed through the CPP in Denmark or its equivalent in the UK [4,5]. This might be due to the frequent occurrence of non-specific symptoms in the general population [6] combined with the low prevalence of OC, implying a low risk of OC when symptoms are presented [7]. This is reflected by low positive predictive values (between 0.2% and 2.5%) for the most frequently reported OC symptoms [7].

Screening trials investigating the effectiveness of Cancer Antigen125 (CA125) and transvaginal ultrasound (TVUS) on asymptomatic women in the general population have shown no effect on OC mortality [8,9]. Hence, early symptom recognition remains the key to earlier diagnosis [3,10]. This calls for introducing direct access (i.e. without first consulting with or referring to a specialist) for GPs to an appropriate investigation. TVUS is considered the first-line examination to detect changes in ovarian structure and size [11]. Screening studies have shown promising results on the diagnostic performance of TVUS for detecting ovarian pathology [12,13]. Yet, TVUS is only available to Danish GPs through CPP referral or waiting list, which often generates months of delay [14].

We aimed to investigate the feasibility of offering direct access to referral to TVUS in a ‘simple evaluation for ovarian cancer’ (SEOC) clinic for women presenting with vague non-specific symptoms in general practice. This included investigating the GP’s referral rate and referral indications, the patient-reported symptoms, and the GP’s subsequent management of test results and findings from the performed TVUS examinations.

## Material and methods

### Study design

We performed a prospective cohort study from 1 April 2017 to 30 April 2018, providing direct access to TVUS for GPs in parts of the Central Denmark Region.

### Setting and GP participants

The tax-funded healthcare system in Denmark offers free access for citizens to medical advice and treatment. GPs are first-line doctors acting as gatekeepers to specialized secondary care, except for emergencies. Hence, access to a gynecologist is only available through referral from general practice. Two SEOC clinics were set up; one at Aarhus University Hospital in April 2017 and another at Randers Regional Hospital in November 2017.

We included 477 GPs in 232 general practices with approx. 190,000 listed women aged ≥40 years during the study period. The GPs were enrolled in municipality-based clusters throughout the study period (Appendix S1).

### Implementation and dissemination

At the beginning of the study and 1 month later, the GPs received an email about the opportunity to request TVUS in the SEOC clinic. The email included a guideline with information about inclusion criteria, referral procedures, how to handle test results, and a list of potential OC symptoms and signs deserving special attention (urinary frequency/urgency, abdominal pain, reduced appetite, irritable bowel syndrome, abdominal bloating and reduced energy). The GPs were instructed to use the referral option as a rule-in test (i.e. a negative TVUS did not exclude OC). Furthermore, if the woman fulfilled the described indications for referral through the CPP for OC, the GP was advised to refer to the CPP.

A third email was sent to the GPs in March 2018 with preliminary results on the use of the SEOC clinics. Additionally, the opportunity to refer to the SEOC clinics was communicated at two meetings for GPs in their local catchment area.

### Patient questionnaire

Guided by an earlier pilot-tested questionnaire [15], we surveyed women before the investigation in the SEOC clinic. A list of 15 symptoms of OC was provided (Appendix S2). Women were asked to register if they had experienced any of the listed symptoms within the past 12 months, including the duration.

### Referral to a SEOC clinic

An electronic referral to a SEOC clinic (including the GP’s indication for referral) was forwarded through the existing online referral system.

### TVUS investigation

Sonographers and nurses performed the TVUS investigations weekly during the study period. They had all undergone theoretical and practical training by gynecological specialists to ensure that the International Ovarian Tumor Analysis (IOTA) Simple Rules were used to assess adnexal masses [16]. In addition, gynecological specialists reviewed the digital images of all identified adnexal masses.

The TVUS was assessed as ‘positive’ if the health professionals identified an ovarian mass, ascites (including postmenopausal with intraperitoneal fluid in the pouch of Douglas), a fibroma (>1 fibroma or fibroma ≥4 cm), an endometrial thickness of >8mm in postmenopausal women, or a tumor in the bladder wall. Uniloculated cysts with a diameter of <4 cm in premenopausal women and of ≤2 cm in postmenopausal women were considered normal findings.

The IOTA Simple Rules [17] were used to categorize ovarian masses into ‘benign’, ‘inconclusive’, or ‘malignant’. Ten features predicted whether the tumor was malignant or benign (Appendix S3). If any M-features were applied (and no B-features), the mass was classified as ‘malignant’. If any B-features were applied (and no M-features), the mass was classified as ‘benign’. However, if both M- and B-features applied or neither M- nor B-features applied, the mass was classified as ‘inconclusive’.

All women with an ovarian mass (benign, malignant, or inconclusive) had additional CA125 testing. Women with an inconclusive or malignant ovarian mass and women with an ovarian mass classified as benign but with an abnormal CA125 (≥35 U/ml) were referred to the CPP.

To ensure that women with ascites were adequately assessed, postmenopausal women with intraperitoneal fluid, even when only located in the pouch of Douglas, were referred to the CPP.

Women with a benign ovarian mass combined with a normal CA125 and postmenopausal women with endometrium of >8 mm without postmenopausal bleeding were referred to a gynecologist within 4 weeks for a repeated TVUS.

Women requiring no further gynecological investigation after a negative TVUS was referred back to the GP, who decided if the additional investigation was needed. After assessment at the SEOC clinic, the GP received an electronic discharge letter including the results of the TVUS and information on any additional hospital-initiated work-up. The GPs were encouraged to consider referral through the CPP if symptoms persisted or worsened.

### Feasibility assessment

The following measures were chosen *a priori* to assess the feasibility of providing GPs with direct access to TVUS (see Appendix S4 for details):

1. Rate of TVUS referral.
2. GP indications for requesting TVUS and patient-reported symptoms/signs prior to referral.
3. Subsequent management within 3 months after a negative TVUS.
4. Findings from TVUS defined as positive (presence of ovarian mass, fibroma, ascites, endometrial thickness, or tumor in the bladder wall) or negative.
5. Major and minor procedures performed within 3 months of TVUS. Major procedures include laparoscopy, laparotomy, and hysterectomy. Minor procedures include endoscopy, curettage, drainage, and excision of tissue.
6. Complications following procedures are defined as reoperation, infection, or death within 1 month of the procedure.
7. Diagnoses after referral to TVUS and positive predictive value for detecting urogynecological cancer. Urogynecological cancer was defined as cancer of the ovary, peritoneum, fallopian tube, endometrium, or bladder (including non-invasive papillary urothelial carcinoma), which are all malignancies detectable by TVUS.

### Data collection

All referrals were registered and linked to registry data through the unique Danish personal identification number. From the patient questionnaires, symptom data was obtained. From the GPs’ electronic referrals, clinical indications, date of referral, and provider number were retrieved. Appendix S5 provides a detailed description of the study data and the data sources, including definitions of variables.

### Statistical analyses

The characteristics of both patients and GPs were described, and the patient-reported duration of symptoms was calculated as medians with interquartile intervals (IQI). The calculation of the women’s propensity for referral was based on the number of referrals per general practice per month per listed 10,000 women aged ≥40 years. The calculation of the practice referral rate was based on the number of referrals per practice per month. Practice groups were compared using Mann–Whitney’s test for continuous data. Pearson’s Chi-squared test was used for nominal data.

We estimated the positive predictive value (including a 95% confidence interval) of identifying urogynecological cancer by using all investigated women with a positive TVUS who needed additional investigation as the denominator. For negative predictive values, all investigated women with a negative TVUS were used as the denominator.

## Results

### Patient characteristics and GP referral rate

**Figure 1.**
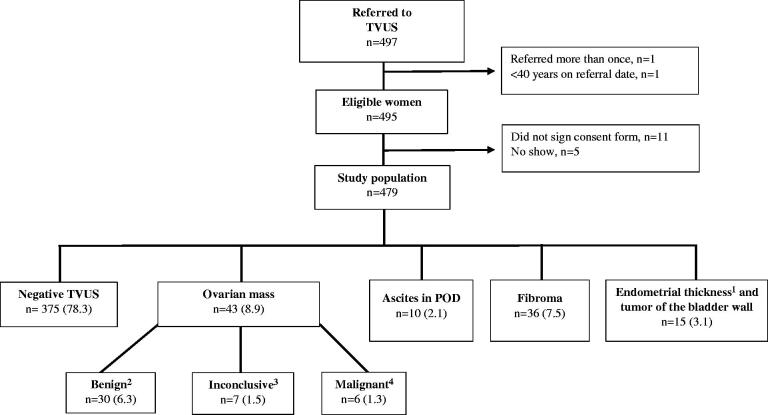
Flowchart of the study population, *n* (%). ¹Endometrial thickness of >8 mm in postmenopausal women. ²A smooth multiloculare cyst <10 cm or a uniloculare cyst ≥4 cm and >2 cm in premenopausal and postmenopausal women, respectively. ³Both M- and B-features present or none of the features present. ^4^Only M-features present. TVUS: transvaginal ultrasound; CPP: cancer patient pathway; OC: ovarian cancer; GP: general practitioner; POD: pouch of Douglas

The inclusion of the 479 participating women is shown in Figure 1. The median age was 58 years (range: 40–89 years), and 67% of the women were postmenopausal (Appendix S6).

During the study period, 232 practices were offered direct access to TVUS investigation, and 120 (51.7%) practices used this opportunity at least once. GPs in single-handed practices were less likely to refer their patients compared to the GPs in practices with more than one GP (*p* < 0.001). The median monthly referral rate per access month was 0.17 (IQI: 0.08–0.34) among all included practices and 0.25 (IQI: 0.10–0.41) among practices requesting TVUS. The median time from referral to the investigation was 7 days (range: 0–35) (Table 1).

**Table 1.** Characteristics of practices requesting direct access to transvaginal ultrasound.

	Included practices	Requested TVUS	Did not request TVUS	*p*-Value
All practices, Number (%)	232	120 (51.7)^a^	112 (48.3)	
Practice type:				
One GP, Number (%)	99 (42.7)	32 (32.3)	67 (67.7)	
≥Two GPs, Number (%)	133 (57.3)	88 (66.2)	45 (33.8)	<0.001^e^
Practice list size of wome*n* ≥ 40 years per GP (median (range))	692 (50–3403)	959 (167–3403)	524 (50–2291)	<0.001^f^
Number of patients referred				
Per GP in practice (median (range))	n/a	2 (1–14)	n/a	
Per practice (median (range))	1 (1–24)	3 (1–24)	0	
Women’s referral rate (median (IQI))^b^	n/a	3.39 (1.84–5.22)	n/a	
Practice referral rate (median (IQI))^c^	0.17 (0.08–0.34)	0.25 (0.10–0.41)	0	
Time (days) from referral to TVUS (median (range))^d^		7 (0–35)		

^a^Eight additional practices referred patients to TVUS. These practices are not included in this table as they were located outside the Central Denmark Region. ^b^Referrals per 10,000 women in GP list (women ≥40 years) per access month. ^c^Referrals per practice per access month. ^d^Expressed in calendar days. ^e^Differences between groups were tested using Pearson’s Chi-squared test. ^f^Differences between groups were tested using Mann–Whitney’s test.

SEOC: simple evaluation for ovarian cancer; TVUS: transvaginal ultrasound; GP: general practitioner; IQI: interquartile interval; n/a: not applicable due to data privacy.

### Indications for referral and patient-reported symptoms

Patient-reported symptoms and GP indications for referring to TVUS are shown in Table 2. The most frequent symptom/indication was lower abdominal/pelvic pain (57.2%), and 39.9% of the patients reported at least three symptoms.

**Table 2.** Symptoms reported by general practitioner and symptoms reported by patient within 12 months of TVUS investigation.

	GP indication for referral			Patient-reported symptoms			
	All referrals (*n* = 479) (%)	Premenopausal (*n* = 158 )	Postmenopausal (*n* = 321)	All (*n* = 397) (%)	Premenopausal (*n* = 134)	Postmenopausal (*n* = 263)	Duration, Median^a^ (IQI)
Abdominal symptoms							
Abdominal pain or discomfort	88 (18.4)	32(20.3)	56 (17.4)	125 (31.5)	47 (35.1)	78 (29.7)	151 (61–365)
Abdominal bloating	94 (19.6)	40 (25.3)	57 (17.8)	231 (58.2)	93 (69.4)	138 (52.5)	200 (85–365)
Increased abdominal size	60 (12.5)	18 (11.4)	42 (13.1)	76 (19.1)	23 (17.2)	53 (20.1)	193 (72–365)
Lower abdominal and pelvic pain	274 (57.2)	97 (61.4)	177 (55.1)	316 (79.6)	112 (83.6)	204 (77.6)	94 (37–302)
Feeling of pressure in the pelvis	110 (23.0)	35 (22.2)	75 (23.4)	14 (3.5)	5 (3.7)	9 (3.4)	48 (21–133)
Abdominal mass	27 (5.6)	9 (5.7)	18 (5.6)	28 (7.1)	11 (8.2)	17 (6.4)	188 (76–365)
Abdominal combined	444 (92.7)	146 (94.3)	298 (92.8)	373 (94.0)	128 (95.5)	245 (93.2)	
Gastrointestinal symptoms							
Constipation	27 (5.6)	8 (5.1)	19 (5.9)	n/a	n/a	n/a	n/a
Diarrhea	14 (2.9)	n/a	10 (3.1)	0	0	0	
Change in bowel habits	n/a	n/a	n/a	102 (25.7)	37 (27.6)	65 (24.7)	144 (47–361)
Indigestion or heartburn	5 (1.0)	n/a	n/a	120 (30.2)	42 (31.3)	78 (29.7)	360 (74–365)
Rectal bleeding	0	0	0	27 (6.8)	16 (11.9)	11 (4.2)	90 (30–360)
Gastrointestinal combined	43 (9.0)	12 (7.6)	31 (9.7)	181 (45.6)	64 (47.8)	117 (44.5)	
Constitutional symptoms							
Weight loss	17 (3.5)	7 (4.4)	10 (3.1)	32 (8.1)	11 (8.2)	21 (8.0)	92 (45–300)
Weight gain	25 (5.2)	9 (5.7)	16 (5.0)	9 (2.3)	n/a	n/a	154 (48–304)
Loss of appetite	11 (2.3)	n/a	n/a	100 (25.2)	35 (26.1)	65 (24.7)	170 (71–365)
Loss of energy	38 (7.9)	14 (8.9)	24 (7.5)	160 (40.3)	60 (44.8)	100 (38.0)	157 (68–365)
Nausea or vomiting	26 (5.4)	9 (5.7)	17 (5.3)	9 (2.3)	n/a	n/a	122 (50–365)
Other constitutional symptoms^b^	n/a	n/a	n/a	0	0	0	
Constitutional combined	89 (18.6)	34 (21.5)	55 (17.1)	206 (51.9)	74 (55.2)	132 (50.2)	
Gynecological symptoms							
Abnormal vaginal bleeding	40 (8.4)	28 (17.7)	12 (3.7)	55 (13.9)	41(30.6)	14 (5.3)	105 (37–321)
Pain during intercourse	26 (5.4)	9 (5.7)	17 (5.3)	84 (21.2)	39 (29.1)	45 (17.1)	252 (86–365)
Other gynecological symptoms^c^	12 (2.5)	n/a	9 (2.8)	n/a	n/a	n/a	n/a
Gynecological combined	76 (15.9)	41 (25.9)	35 (10.9)	121 (30.5)	66 (49.3)	55 (20.9)	
Urinary tract symptoms							
Urinary frequency	52 (10.9)	16 (10.1)	36 (11.2)	193 (48.6)	68 (50.7)	125 (47.5)	165 (47–365)
Urinary urgency	11 (2.3)	n/a	n/a	155 (39.0)	44 (32.8)	111 (42.2)	218 (62–365)
Other urinary tract symptoms^d^	20 (4.2)	7 (4.4)	13 (4.0)	8 (2.0)	n/a	n/a	n/a
Urinary combined	80 (16.7)	26 (16.5)	54 (16.8)	236 (59.4)	77 (57.5)	159 (60.5)	
Other symptoms and signs							
Back pain	32 (6.7)	10 (6.3)	22 (6.9)	107 (27.0)	36 (26.9)	71 (27.0)	120 (24–365)
Other symptoms^e^	21 (4.4)	6 (3.8)	15 (4.7)	43 (10.8)	13 (9.7)	30 (11.4)	246 (165–326)
Multiple symptoms and signs^f^							
Did not respond to the questionnaire	0 (0)	0 (0)	0 (0)	82 (17.1)	24 (15.2)	58 (18.1)	
1 symptom	148 (30.9)	43 (27.2)	105 (32.7)	25 (5.2)	5 (3.2)	20 (6.2)	
2 symptoms	140 (29.2)	46 (29.1)	94 (29.3)	42 (8.8)	11 (6.9)	31 (9.7)	
≥3 symptoms	191 (39.9)	69 (43.7)	122 (38.0)	330 (68.9)	118 (74.7)	212 (66.0)	

*Note*. Total median duration of patient symptoms 154 days (IQI: 50–365 days) (*n* = 479).

^a^Duration in days. Information provided by the patient. Some missing data. ^b^Other constitutional symptoms; GP indication: impaired general condition. Patient-reported: none. ^c^Other gynecological symptoms; GP indication: vaginal discharge, cyst on MR/CT and enlarged uterus. Patient-reported: vaginal discharge. ^d^Other urinary tract symptoms; GP indication: nocturia, dysuria, hematuria, urinary incontinence and urination symptoms. Patient-reported: hematuria, urinary incontinence and dysuria. ^e^Other symptoms; GP indication: genetic predisposition, patient concerned about OC, GP’s gut feeling, dyspnea, edema, anemia and hot flushes. Patient-reported: dyspnea, edema, hot flushes, dizziness, night sweats, discomfort in lower extremities, palpitations, and globus sensation. ^f^Percentages are calculated based on the entire population, *n* = 479.

*n*: number of patients; IQI: interquartile interval; GP: general practitioner; OC: ovarian cancer; n/a: not applicable (due to data privacy because of low numbers).

A total of 397 (82.9%) women filled in the questionnaire before visiting the SEOC clinic. The most frequently reported symptoms were lower abdominal/pelvic pain (79.6%) and abdominal bloating (58.2%). A total of 83.1% of the women reported to have experienced at least three symptoms within the past year; the median duration varied from 48 to 360 days.

### Findings from TVUS, performed procedures, and procedure-related complications

Information on procedures, diagnoses, and subsequent management of included women is shown in Table 3. Of the 479 women undergoing TVUS, 104 (21.7%) had a positive TVUS. A total of 68 (14.2%) of these women needed additional investigations; seven (6.7%) underwent major surgery, and 21 underwent minor procedures. All major surgical procedures resulted in histologically verified pathology or were performed due to vaginal prolapse; this confirmed the findings of TVUS. No complications were registered after surgery.

**Table 3.** Contact to the GPs, diagnostic investigations, procedures and diagnoses within 3 months of transvaginal ultrasound in a SEOC clinic.

	Total TVUS, *n* (%)	Negative TVUS, *n* (%)	Positive TVUS, *n* (%)
Number of women	479 (100.0)	375 (78.3)	104 (21.7)
Contact to GP^a^	413 (86.2)	323 (86.1)	90 (86.5)
Referrals			
CPP (any)	22 (4.6)	16 (4.3)	6 (5.7)^1^
Gynecologist	52 (10.9)	37 (9.9)	15 (14.4)^2^
Abdominal ultrasound	26 (5.4)	15 (4.0)	11 (10.6)
MRI	26 (5.4)	14 (3.7)	12 (11.5)
Procedures			
Major^b^	n/a	n/a	7 (6.7)
Minor^c^	31 (6.5)	10 (2.7)	21 (20.2)
No subsequent management^d^	52 (10.9)	47 (12.5)	5 (4.8)^2^
Diagnoses			
Ovarian cancer	0	0	0
Urogynecological cancer^e^	3 (0.6)	0	3 (2.9)
Other cancer and precancerous lesion^f^	6 (1.3)	6 (1.6)	0

^1^Not initiated as part of the TVUS algorithm. ^2^Includes only fibromas.

^a^Including face-to-face consultations, telephone consultations, and email consultations. ^b^Laparotomy, laparoscopy and hysterectomy. These procedures led to the diagnosis of either cysts, fibromas, vaginal prolapse, or urogynecological cancer. ^c^Endoscopy, curettage of the uterus, drainage of ascites, drainage of abscess, and excision of pathological tissue. ^d^No contact to the GP, or not referred to a CPP, gynecologist, abdominal ultrasound, gastroscopy, colonoscopy, MRI, or no performed procedure. ^e^Includes cancer of the endometrium or bladder (including non-invasive papillary urothelial carcinoma). ^f^Includes cancer of, for example, the intestines or kidneys (all cases were non-detectable by TVUS). Due to data protection regulations, it is not possible to provide data on the specific cancer findings due to the low numbers.

TVUS: transvaginal ultrasound; SEOC: simple evaluation for ovarian cancer; GP: general practitioner; CPP: cancer patient pathway; MRI: magnetic resonance imaging; n/a: not applicable due to data protection for <3 observations.

### Subsequent management after a negative TVUS

Among the 375 women with a negative TVUS, 323 (86.1%) consulted their GP within 3 months. Of these women, 241 (74.6%) were managed in general practice without subsequent referral. A total of 47 (12.5%) received no subsequent management (Table 3).

### Diagnoses and positive predictive value for detecting urogynecological cancer

Three cases of urogynecological cancer were diagnosed (7–50 days after TVUS) in women with a positive TVUS, which yielded a positive predictive value of 4.4% (95% confidence intervals: 1.5–12.2) (Table 4). Additionally, six women with a negative TVUS were diagnosed with cancer or a precancerous lesion during the 3-month follow-up according to the Danish Pathology Register (Table 3). Extending the follow-up period to 6 months resulted in two additional cancer diagnoses in women with a negative TVUS. All eight malignancies were located outside the urogenital organs and could not be diagnosed by TVUS.

**Table 4.** Referrals to TVUS in a SEOC clinic, positive TVUS with additional investigations needed, and diagnosed urogynecological cancers.

	Requested TVUS, *n* (%)	Positive TVUS^a^, *n* (%)	Positive TVUS requiring additional investigations^b^, *n* (%)	Urogynecological cancer after TVUS and requiring additional investigations, *n*	Rate of additional investigations after TVUS request, % (95% CI)	PPV for urogynecological cancer after TVUS request, PPV (95% CI)	NPV for urogynecological cancer after TVUS request, NPV (95% CI)
All women	479 (100.0)	104 (21.7)	68 (14.2)	3	14.2 (11.4–17.6)	4.4 (1.5–12.2)	100 (99.1–100.0)
Postmenopausal	321 (67.0)	79 (24.6)	56 (17.4)	3	17.4 (13.7–22.0)	5.4 (1.8–14.6)	100 (98.6–100.0)
Premenopausal	158 (33.0)	25 (15.8)	12 (7.6)	0	7.6 (4.4–12.8)	0.0	0.0

^a^Includes: fibromas (*n* = 36), ovarian masses (*n* = 43), ascites in POD (*n* = 10), endometrial thickness and bladder abnormalities (*n* = 15). ^b^Includes: positive TVUS except fibroma.

SEOC: simple evaluation for ovarian cancer; TVUS: transvaginal ultrasound; PPV: positive predictive value; NPV: negative predictive value; CI: confidence interval; POD: pouch of Douglas.

## Discussion

We assessed the feasibility of offering GPs direct access to a SEOC clinic for women presenting with vague non-specific symptoms of potential OC. The number of referrals to TVUS was low (an average of three annual referrals per practice). However, only half of the enrolled practices used the opportunity to refer to TVUS during the study period.

Lower abdominal/pelvic pain was the most frequently reported symptom (by 80% of women) prompting referral to TVUS at a SEOC clinic (by 57% of GPs). A positive TVUS result was identified in 104 (21.7%) women. Three (0.6%) were diagnosed with urogynecological cancer, and seven (1.5%) underwent major surgery; all without complications. Offering TVUS as a rule-in test for OC in symptomatic women seen in general practice was feasible; OC was excluded in the majority of women without introducing surgical complications from false-positive results.

### Strengths and limitations

An important strength of our study is that it examines how nurses and sonographers manage TVUS (using the IOTA Simple Rules) in symptomatic women. Evidence from previous studies suggests that using the IOTA Simple Rules may be superior to using the Risk of Malignancy Index (RMI), particularly in premenopausal women [18,19], and that the IOTA Simple Rules perform well in the hands of less experienced examiners, for example, sonographers [18,20]. This was supported by our study, as no woman had a false negative test.

TVUS holds a risk of generating false positives, which may lead to repeated TVUS, CA125 testing, or even unnecessary surgery [21]. In this study, all TVUS-related procedures were performed on symptomatic women with vaginal prolapse or with histologically confirmed pathology in the bladder, uterus, or ovaries. All procedures were performed without reported complications.

Eight women (2.1%) with a negative TVUS were diagnosed with cancer outside the urogenital organs during the 6-month follow-up, which supports that cancer symptoms often evolve over time as cancer grows. This underlines the importance of providing GPs with the option to refer women with vague non-specific symptoms to relevant diagnostic investigations in order to reduce diagnostic delay. Furthermore, it emphasizes the importance of subsequent GP follow-up when access to a rule-in test is provided.

Only 51.7% of the included practices used the opportunity to refer to TVUS. This could indicate barriers for the use, especially among single-handed practices, as their GPs were significantly less likely to use the opportunity to refer. However, the number of referrals increased at the end of the study (data not shown). This suggests that an adaption period should be expected after implementation.

The study is generalizable to similar healthcare settings with GPs acting as gatekeepers, and the reported findings can be used to facilitate the implementation of direct access referral routes in general practice for cancers that do not fulfil the criteria for referral through the CPPs.

### Comparison to existing literature

Two large prospective studies support the value of rapid evaluation of women presenting symptoms of potential OC [22,23]. A US study assessed the value of using a symptom index to select women for investigation with TVUS and CA125. A higher proportion of early-stage OCs were diagnosed than what was expected from national statistics. The authors suggested that the symptom index might act as an educational tool by increasing the awareness of symptoms and prompting the women to seek care early [23]. In the DOvE study, women were evaluated through testing by TVUS and serial CA125. No evidence of change in stage distribution was identified. However, interestingly, included women had a lower tumor burden compared to women diagnosed through usual care [22]. As complete tumor resection is the key prognostic factor for disease survival [24], the true value of symptom-based assessment could be to identify OC when tumor resection is still possible rather than to produce a stage shift. Due to differences in the applied methods and inclusion criteria, the findings of the present study cannot be compared directly to the findings of these studies. First, the previous studies [22,23] performed CA125 testing in all women undergoing TVUS. Second, in both studies, TVUS was performed by experienced investigators. Third, the OC incidence must be assumed to be higher than in the present study as both previous studies included women referred due to strong GP suspicion of OC. Consistent with these studies, our study calls for further exploration of the benefits and harms of offering prompt symptom-based interventions to potential OC patients.

A common criticism of direct access through general practice is that it might increase the number of inappropriate referrals without improving the diagnostic yield [25]. In line with a recent review of direct access to cancer testing in general practice [26], our study suggests that these concerns are unsupported as the use was low. Most women were postmenopausal, and more than 20% of the women referred to TVUS were diagnosed with a clinically relevant finding that is likely to explain their symptoms (most often lower abdominal/pelvic pain). This is in accordance with two case-control studies, which identified abdominal pain as the most frequent patient-reported OC symptom [7,27].

When GPs refer women through the CPP, approx. one in ten is diagnosed with OC [4]. In the present study, one in five women had a positive TVUS, and one in seven needed further investigation. This strongly suggests that offering GPs direct access to TVUS did not increase the number of inappropriate referrals.

Several possible benefits exist from offering direct access to TVUS. These include the potential of reducing healthcare costs by efficient use of available resources [28], facilitating more timely diagnosis, and increasing both patient and GP satisfaction. This is supported in two studies reporting high patient and GP satisfaction from ensuring direct access [28,29] and high patient acceptability of TVUS and CA125 testing when symptoms are present [30]. Therefore, it is reasonable to assume that the same high satisfaction applies to the present study.

## Conclusion

This study is the first to investigate the feasibility of offering direct access from general practice to TVUS at a SEOC clinic for women presenting with vague non-specific symptoms of potential OC. Our findings support the feasibility and indicate a possible benefit of implementing SEOC clinics. However, it remains unanswered whether it will lead to improved outcome in women with OC, and this should be investigated in future large-scale studies.

## Supplementary Material

Supplemental Material
