# Supplementary material for: Direct access from general practice to transvaginal ultrasound for early detection of ovarian cancer: a feasibility study
Source: Scand J Prim Health Care. 2021 Jun 7;39(2):230–9. doi: 10.1080/02813432.2021.1922831 (PMC8293964; doi:10.1080/02813432.2021.1922831)
Supplement: Supplemental Material [file IPRI_A_1922831_SM6125.docx]

**Supplementary**

**Appendix S1.** *Date of inclusion of practice clusters throughout the 13-month study period*


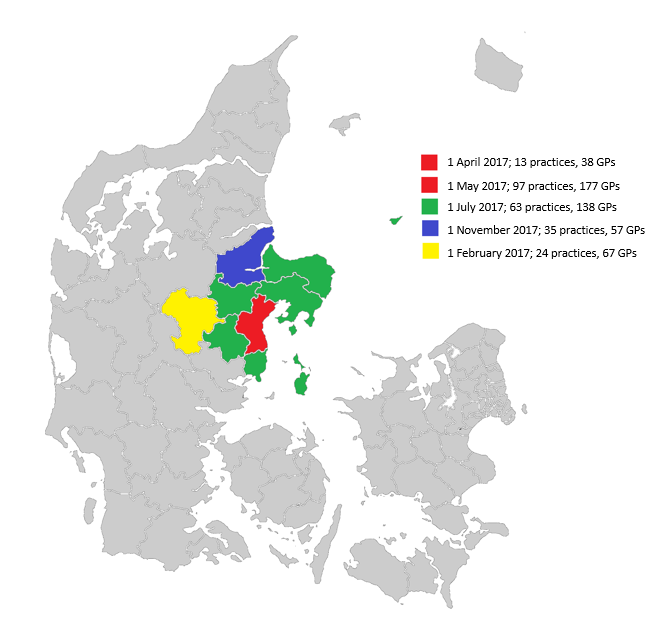


| Able to feel an abdominal mass |
| --- |
| Continuous abdominal distension |
| Occasional bloating |
| Heartburn or indigestion |
| Pain in upper part of abdomen |
| Pain in lower part of abdomen and/or pelvis |
| Reduced appetite or early satiety |
| Urinary frequency |
| Urinary urgency |
| Change in bowel habits |
| Abnormal vaginal bleeding |
| Pain during intercourse |
| Rectal bleeding |
| Loss of weight |
| Fatigue |
| Other symptom |

**Appendix S2.** Symptoms included in the patient questionnaire

| Able to feel an abdominal mass |
| --- |
| Continuous abdominal distension |
| Occasional bloating |
| Heartburn or indigestion |
| Pain in upper part of abdomen |
| Pain in lower part of abdomen and pelvis |
| Reduced appetite or early satiety |
| Urinary frequency |
| Urinary urgency |
| Change in bowel habits |
| Abnormal vaginal bleeding |
| Pain during intercourse |
| Rectal bleeding |
| Loss of weight |
| Fatigue |
| Other |

**Appendix S1**Symptoms included in the patient questionnaire2

**Appendix S2.** Symptoms included in the patient questionnaire

| Able to feel an abdominal mass |
| --- |
| Continuous abdominal distension |
| Occasional bloating |
| Heartburn or indigestion |
| Pain in upper part of abdomen |
| Pain in lower part of abdomen and pelvis |
| Reduced appetite or early satiety |
| Urinary frequency |
| Urinary urgency |
| Change in bowel habits |
| Abnormal vaginal bleeding |
| Pain during intercourse |
| Rectal bleeding |
| Loss of weight |
| Fatigue |
| Other |

**Appendix S1**Symptoms included in the patient questionnaire2

| **Appendix S3.** Malignant and benign features of the IOTA ”Simple Rules” | |
| --- | --- |
| Rules for predicting a benign tumor  (B-features) | Rules for predicting a malignant tumor  (M-features) |
| B1 Uniloculare cyst | M1 Irregular solid tumor |
| B2 Presence of solid components for  nnnnwhich the largest solid component  nnnnis < 7 mm in the largest diameter | M2 Presence of ascites |
| B3 Presence of acoustic shadows | M3 At least four papillary structures |
| B4 Smooth multilocular tumor with  nnnnlargest diameter < 100 mm | M4 Irregular multilocular solid tumor   nnnnwith largest diameter ≥ 100 mm |
| B5 No blood flow on Doppler | M5 Very strong blood flow on Doppler |

**Appendix S4.** Feasibility assessment - measures and definitions

| **Measure** | **Definition** |
| --- | --- |
| Rate of TVUS referral | Practice referral rate was based on number of referrals per practice per month. |
| GP indications for requesting TVUS and patient-reported symptoms and signs before TVUS | Only patients who filled in the questionnaire before TVUS were included in the analyses. Symptom duration exceeding 12 months was truncated at 365 days. |
| Subsequent management within 3 months after a negative TVUS (no additional investigation was needed as part of study participation, and these women were referred back to their GP) | Subsequent management was defined as contact with the GP, referral to a CPP (not only OC), referral to a gynecologist, abdominal ultrasound, MRI, or endoscopy.  Contact with the GP was defined as face-to-face, email, or telephone consultation.  Endoscopy was defined as hysteroscopy, colposcopy, cystoscopy, colonoscopy, sigmoidoscopy, or gastroscopy. |
| Findings from TVUS in the SEOC clinic | Defined as negative or positive (presence of ovarian mass, fibroma, ascites (including intraperitoneal fluid in the pouch of Douglas in postmenopausal women), endometrial thickness, or tumor in the bladder wall). |
| Major and minor procedures performed within 3 months of TVUS in the SEOC clinic | Major procedures were defined as laparoscopy and laparotomy, including hysterectomy.  Minor procedures were defined as endoscopy, curettage, drainage, and excision of tissue. |
| Complications following procedures | Defined as reoperation, infection, or death within one month of the procedure. |
| Diagnoses after requesting TVUS at a SEOC clinic and positive predictive value for detecting urogynecological cancer | Urogynecological cancers and precancerous lesions diagnosed within three and six months of TVUS were included. Urogynecological cancer was defined as cancer of the ovary, peritoneum, fallopian tube, endometrium, or bladder (including non-invasive papillary urothelial carcinoma), which are all malignancies that are detectable with TVUS.  Relevant histological findings identified by surgery were defined as fibroma of the ovaries and/or fallopian tubes, fibroma of the uterus, ovarian cyst, endometrial hyperplasia, endometrial cancer, and urothelial papilloma or atypia. |

| **Study data** | **Data sources and definition** |
| --- | --- |
| **Symptom data** | Symptom data was retrieved from the patient questionnaire. |
| **Clinical indications for referral, date of referral, and provider number** | Data on clinical indications, date of referral, and provider number was collected from the GPs’ electronic referrals. |
| **Practice population in each general practice** | Practice population was retrieved from the patient lists. |
| **Educational level** | Data on educational level (<10 years, 10-15 years, >15 years) was retrieved from Statistics Denmark. |
| **Marital status** | Marital status (married/cohabitating, single) was retrieved from Statistics Denmark. |
| **Ethnic origin** | Data on ethnic origin (Danish, immigrant) was retrieved from Statistics Denmark. |
| **Charlson Comorbidity Index (CCI)** | CCI was calculated using 10-year history before TVUS in the SEOC clinic minus 1 day, divided into 3 groups (0, 1-2, and ≥3)¹. |
| **TVUS results** | TVUS results were entered into an online secure database (RedCap) directly after the investigation. |
| **Patient age** | Do. |
| **Menopausal status** | Do. |
| **Previous cancers** | Previous cancers (except non-melanoma skin cancer (ICD-10: C44) were obtained from the Danish Cancer Registry (DCR)². |
| **Investigations performed at a private or public hospital after TVUS** | These investigations were identified from the Danish National Patient Registry (DNPR)³. |
| **Procedures** | Procedures were identified in the DNPR. |
| **Complications** | Complications were identified in the DNPR. |
| **Private practicing specialist services (including GPs)** | The Danish National Health Service Register was used to collect data on these services⁴. |
| **Cancer diagnoses and precancerous lesions** | These diagnoses were retrieved from the Danish Pathology Register⁵. |

**Appendix S5.** Study data, data sources, and definitions

*¹Quan H, Li B, Couris CM, Fushimi K, Graham P, Hider P, et al. Updating and validating the Charlson comorbidity index and score for risk adjustment in hospital discharge abstracts using data from 6 countries. Am J Epidemiol. 2011;173(6):676-82.
²Gjerstorff ML. The Danish Cancer Registry. Scand J Public Health. 2011;39(7 Suppl):42-5.
³Lynge E, Sandegaard JL, Rebolj M. The Danish National Patient Register. Scand J Public Health. 2011;39(7 Suppl):30-3.
⁴Andersen JS, Olivarius Nde F, Krasnik A. The Danish National Health Service Register. Scand J Public Health. 2011;39(7 Suppl):34-7.
⁵Bjerregaard B, Larsen OB. The Danish Pathology Register. Scand J Public Health. 2011;39(7 Suppl):72-4.*

**Appendix S2**. Study data, data sources and definition

**Appendix S6.** Characteristics of the 479 patients referred to direct access transvaginal ultrasound from general practice

|  | **Patients referred (n=479)** |
| --- | --- |
| **Median age (IQI)** | 58 (49-67) |
| **Age (years)** |  |
| 40-59 | 267 (55.7%) |
| ≥60 | 212 (44.3%) |
| **Menopausal status¹** |  |
| Premenopausal | 158 (33.0) |
| Postmenopausal | 321 (67.0) |
| **Educational level** |  |
| <10 years | 18.4 |
| 10-15 years | 40.5 |
| >15 years | 39.3 |
| Unknown | 1.9 |
| **Comorbidity** |  |
| CCI=0 | 432 (90.2%) |
| CCI=1-2 | 41 (8.6%) |
| CCI=≥3 | 6 (1.3%) |
| **Ethnicity** |  |
| Danish | 91.4% |
| Immigrant | 8.4% |
| Unknown | 0.2% |
| **Marital status** |  |
| Married/cohabitating | 62.0% |
| Living alone | 37.8% |
| Unknown | 0.2% |
| **Previous cancer²** |  |
| ≥ 10 years | 12 (2.5%) |
| < 10 years | 20 (4.2%) |

¹Menopausal status was defined as no vaginal bleeding for more than one year or hysterectomy and age ≥50 years.
²Registered in the Danish Cancer Register or the Danish National Patient Register with a cancer diagnosis (except non-melanoma skin cancer (C44)) before study start (either ≥10 years or within 10 years).
